# 5-Methyl-3,3-bis­(morpholin-4-yl)-1-[2-(morpholin-4-yl)eth­yl]-2,3-dihydro-1*H*-indol-2-one

**DOI:** 10.1107/S1600536812007155

**Published:** 2012-02-24

**Authors:** Hui-Hui Lin, Wei-Yao Wu, Jing-Jing Zhang, Sheng-Li Cao

**Affiliations:** a Department of Chemistry, Capital Normal University, Beijing 100048, People’s Republic of China

## Abstract

In the title compound, C_23_H_34_N_4_O_4_, the morpholine rings adopt chair conformations. The N atom of the indol-2-one group is linked to the N atom of one morpholine ring through a flexible ethyl group with an almost *cif* conformation. In the crystal, molecules are linked by C—H⋯O interactions into infinite chains along the *c* direction. The almost parallel infinite chains are further inter­connected *via* other sets of C—H⋯O inter­actions, forming a three-dimensional framework.

## Related literature

For background to and activities of indoline-2,3-dione and its derivatives, see Chiyanzu *et al.* (2005 ▶); Karali (2002 ▶); Sirisoma *et al.* (2009 ▶); Solomon *et al.* (2009 ▶); Sriram *et al.* (2004 ▶). For structural analogues of indoline-2,3-dione (isatin), see: Wang *et al.* (2012 ▶).
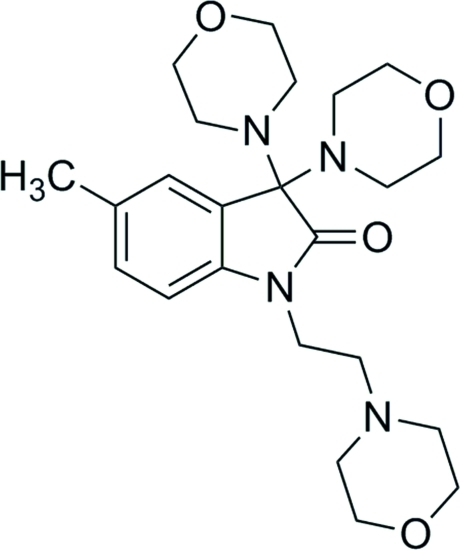


## Experimental

### 

#### Crystal data

C_23_H_34_N_4_O_4_
*M* *_r_* = 430.54
Monoclinic, 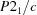

*a* = 10.1772 (10) Å
*b* = 14.2576 (14) Å
*c* = 15.8950 (16) Å
β = 97.332 (2)°
*V* = 2287.5 (4) Å^3^
*Z* = 4
Mo *K*α radiation
μ = 0.09 mm^−1^
*T* = 296 K
0.40 × 0.30 × 0.20 mm

#### Data collection

Bruker APEXII CCD diffractometer
Absorption correction: multi-scan (*SADABS*; Bruker, 2007 ▶) *T*_min_ = 0.660, *T*_max_ = 0.746
12380 measured reflections
5355 independent reflections
2599 reflections with *I* > 2σ(*I*)
*R*_int_ = 0.040

#### Refinement

*R*[*F* ^2^ > 2σ(*F* ^2^)] = 0.056
*wR*(*F* ^2^) = 0.141
*S* = 1.01
5355 reflections
280 parameters
H-atom parameters constrained
Δρ_max_ = 0.18 e Å^−3^
Δρ_min_ = −0.16 e Å^−3^

### 

Data collection: *APEX2* (Bruker, 2007 ▶); cell refinement: *APEX2* and *SAINT* (Bruker, 2007 ▶); data reduction: *SAINT*; program(s) used to solve structure: *SHELXS97* (Sheldrick, 2008 ▶); program(s) used to refine structure: *SHELXL97* (Sheldrick, 2008 ▶); molecular graphics: *SHELXTL* (Sheldrick, 2008 ▶); software used to prepare material for publication: *SHELXTL* and *PLATON* (Spek, 2009 ▶).

## Supplementary Material

Crystal structure: contains datablock(s) I, global. DOI: 10.1107/S1600536812007155/zq2153sup1.cif

Structure factors: contains datablock(s) I. DOI: 10.1107/S1600536812007155/zq2153Isup2.hkl

Supplementary material file. DOI: 10.1107/S1600536812007155/zq2153Isup3.cml

Additional supplementary materials:  crystallographic information; 3D view; checkCIF report

## Figures and Tables

**Table 1:** Hydrogen-bond geometry (Å, °)

*D*—H⋯*A*	*D*—H	H⋯*A*	*D*⋯*A*	*D*—H⋯*A*
C20—H20*B*⋯O3^i^	0.97	2.62	3.109 (3)	112
C15—H15*A*⋯O3^ii^	0.97	2.52	3.480 (2)	173
C17—H17*A*⋯O2^iii^	0.97	2.66	3.395 (3)	132
C13—H13*B*⋯O4^iv^	0.97	2.64	3.318 (3)	128

Symmetry codes: (i) 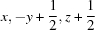; (ii) 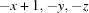; (iii) 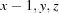; (iv) 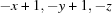.

**Fig. 1.**
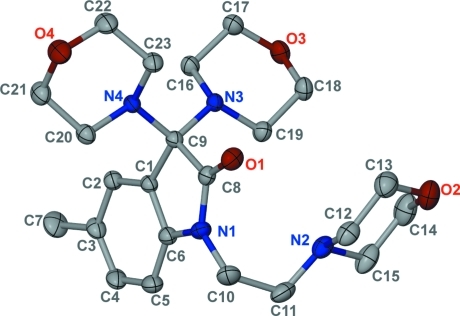
The title molecule with the atomic numbering scheme. The displacement ellipsoids of the non-H atoms are shown at the 30% probability level. All H atoms are omitted for clarity.

**Fig. 2.**
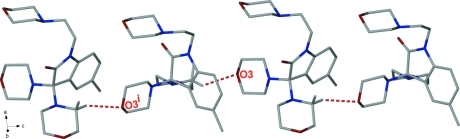
The array of the title compound along the c direction. The red-dashed lines indicate C—H···O interactions. H atoms are omitted for clarity, except those involved in bonding interactions.

**Fig. 3.**
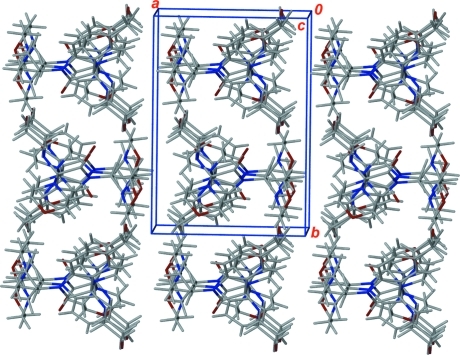
View down the c direction of the stacking structure of the title compound.

## supplementary crystallographic information

### Comment

Mannich base derivatives of indoline-2,3-dione (isatin) were reported having a wide range of biological activities such as antibacterial (Chiyanzu *et al.*, 2005), anti-HIV (Sriram *et al.*, 2004) and anticancer activity (Karali, 2002; Sirisoma *et al.*, 2009; Solomon *et al.*, 2009). To obtain isatin Mannich base analogues with a flexible ethylene linker between the N atom of the isatin and the amine group of the morpholine, we designed the reaction of 1-(2-bromoethyl)-5-methylindoline-2,3-dione with an excess of morpholine. Interestingly, an additional nucleophilic addition of morpholine to one carbonyl group on the isatin simultaneously occurred to give the title compound (Scheme 1), which might also exhibit potential antitumor properties. Herein, we report the crystal structure of this new compound.

In the title compound, C_23_H_34_N_4_O_4_, the morpholine rings adopt chair conformations. The N1 atom of the indol-2-one moiety is linked to the N2 atom of a morpholine ring through a flexible ethylene group with an almost *cis* conformation [the torsion angle N1—C10—C11—N2 is 59.7 (3)°, as shown in Fig. 1]. Such a fashion is different from the *trans* conformation [corresponding torsion angle of 175.74 (11)°] found in 2-(5-fluoro-2,3-dioxoindolin-1-yl)ethyl 4-methylpiperazine-1-carbodithioate reported by us recently (Wang *et al.*, 2012). The two morpholine groups at C9 (*sp*^3^) exhibit a N4—C9—N3 bond angle of 107.45 (15)°. Along the *c* axis, the molecules are interconnected and arranged into arrays through intermolecular C20—H20B(methylene)···O3^i^(ether) interactions (see Table 1 and Fig. 2). The almost parallel arrays formed are further interconnected *via* other sets of C—H(methylene)···O(ether) interactions (Table 1), forming a three-dimensional framework (Fig. 3).

### Experimental

To a solution of 1-(2-bromoethyl)-5-methylindoline-2,3-dione (0.27 g, 1 mmol) in *N*,*N*-dimethylformamide (5 ml) was added morpholine dropwise (0.52 g, 6 mmol). The mixture was stirred at 80–90°C for 3 h. The colourless crystals of the title compound were deposited by evaporation of the resulting solution at room temperature for one day (m.p. 450.2–451.9 K; yield 53%).

### Refinement

All H atoms were discernible in the difference electron density maps. Nevertheless, the H atoms were placed into idealized positions and allowed to ride on their respective carrier atoms, with C—H = 0.93 and *U*_iso_(H) = 1.2*U*_eq_(C) for aromatic H atoms, with C—H = 0.97 and *U*_iso_(H) = 1.2*U*_eq_(C) for methylene H atoms, and with C—H = 0.96 and *U*_iso_(H) = 1.5*U*_eq_(C) for methyl H atoms.

### Crystal data

**Table:**

C_23_H_34_N_4_O_4_	*Z* = 4
*M**_r_* = 430.54	*F*(000) = 928
Monoclinic, *P*2_1_/*c*	*D*_x_ = 1.250 Mg m^−^^3^
Hall symbol: -P 2ybc	Mo *K*α radiation, λ = 0.71073 Å
*a* = 10.1772 (10) Å	µ = 0.09 mm^−^^1^
*b* = 14.2576 (14) Å	*T* = 296 K
*c* = 15.8950 (16) Å	Block, colourless
β = 97.332 (2)°	0.40 × 0.30 × 0.20 mm
*V* = 2287.5 (4) Å^3^	

### Data collection

**Table:**

Bruker APEXII CCD diffractometer	5355 independent reflections
Radiation source: fine-focus sealed tube	2599 reflections with *I* > 2σ(*I*)
Graphite monochromator	*R*_int_ = 0.040
ω scans	θ_max_ = 28.0°, θ_min_ = 1.9°
Absorption correction: multi-scan (*SADABS*; Bruker, 2007)	*h* = −13→10
*T*_min_ = 0.660, *T*_max_ = 0.746	*k* = −18→14
12380 measured reflections	*l* = −20→20

### Refinement

**Table:**

Refinement on *F*^2^	Primary atom site location: structure-invariant direct methods
Least-squares matrix: full	Secondary atom site location: difference Fourier map
*R*[*F*^2^ > 2σ(*F*^2^)] = 0.056	Hydrogen site location: inferred from neighbouring sites
*wR*(*F*^2^) = 0.141	H-atom parameters constrained
*S* = 1.01	*w* = 1/[σ^2^(*F*_o_^2^) + (0.0533*P*)^2^ + 0.0135*P*] *P* = (*F*_o_^2^ + 2*F*_c_^2^)/3
5355 reflections	(Δ/σ)_max_ < 0.001
280 parameters	Δρ_max_ = 0.18 e Å^−^^3^
0 restraints	Δρ_min_ = −0.16 e Å^−^^3^

### Special details

**Table:**

Geometry. All e.s.d.'s (except the e.s.d. in the dihedral angle between two l.s. planes) are estimated using the full covariance matrix. The cell e.s.d.'s are taken into account individually in the estimation of e.s.d.'s in distances, angles and torsion angles; correlations between e.s.d.'s in cell parameters are only used when they are defined by crystal symmetry. An approximate (isotropic) treatment of cell e.s.d.'s is used for estimating e.s.d.'s involving l.s. planes.
Refinement. Refinement of *F*^2^ against ALL reflections. The weighted *R*-factor *wR* and goodness of fit *S* are based on *F*^2^, conventional *R*-factors *R* are based on *F*, with *F* set to zero for negative *F*^2^. The threshold expression of *F*^2^ > σ(*F*^2^) is used only for calculating *R*-factors(gt) *etc*. and is not relevant to the choice of reflections for refinement. *R*-factors based on *F*^2^ are statistically about twice as large as those based on *F*, and *R*-factors based on ALL data will be even larger.

### Fractional atomic coordinates and isotropic or equivalent isotropic displacement parameters (Å^2^)

**Table:**

	*x*	*y*	*z*	*U*_iso_*/*U*_eq_	
N1	0.58843 (17)	0.24574 (12)	0.17404 (11)	0.0495 (5)	
N2	0.80352 (17)	0.17783 (11)	0.08141 (13)	0.0561 (5)	
N3	0.36653 (15)	0.22400 (10)	−0.00090 (9)	0.0398 (4)	
N4	0.29148 (16)	0.34238 (10)	0.08565 (9)	0.0404 (4)	
O1	0.58871 (14)	0.35530 (10)	0.06837 (9)	0.0577 (4)	
O2	0.90210 (18)	0.16969 (14)	−0.07774 (12)	0.0859 (6)	
O3	0.31081 (16)	0.09322 (10)	−0.13528 (9)	0.0579 (4)	
O4	0.17921 (17)	0.52235 (10)	0.10253 (10)	0.0706 (5)	
C1	0.3731 (2)	0.19469 (12)	0.15619 (12)	0.0404 (5)	
C2	0.2663 (2)	0.14541 (13)	0.17824 (12)	0.0460 (5)	
H2A	0.1832	0.1533	0.1471	0.055*	
C3	0.2818 (2)	0.08355 (13)	0.24716 (14)	0.0533 (6)	
C4	0.4063 (3)	0.07408 (15)	0.29203 (14)	0.0619 (7)	
H4A	0.4176	0.0328	0.3377	0.074*	
C5	0.5156 (3)	0.12359 (16)	0.27181 (14)	0.0608 (6)	
H5A	0.5987	0.1160	0.3029	0.073*	
C6	0.4963 (2)	0.18449 (13)	0.20389 (13)	0.0460 (5)	
C7	0.1639 (3)	0.03148 (16)	0.27269 (16)	0.0784 (8)	
H7A	0.1921	−0.0078	0.3207	0.118*	
H7B	0.1253	−0.0066	0.2262	0.118*	
H7C	0.0995	0.0757	0.2874	0.118*	
C8	0.5323 (2)	0.29554 (14)	0.10519 (13)	0.0440 (5)	
C9	0.38464 (19)	0.26432 (12)	0.08495 (12)	0.0389 (5)	
C10	0.7280 (2)	0.25298 (17)	0.20717 (15)	0.0660 (7)	
H10A	0.7376	0.2489	0.2686	0.079*	
H10B	0.7611	0.3137	0.1922	0.079*	
C11	0.8101 (2)	0.17647 (17)	0.17284 (16)	0.0702 (7)	
H11A	0.9018	0.1836	0.1975	0.084*	
H11B	0.7795	0.1160	0.1903	0.084*	
C12	0.8768 (2)	0.25529 (14)	0.05026 (17)	0.0640 (7)	
H12A	0.9702	0.2493	0.0712	0.077*	
H12B	0.8452	0.3142	0.0707	0.077*	
C13	0.8582 (3)	0.25463 (18)	−0.04466 (18)	0.0795 (8)	
H13A	0.7651	0.2636	−0.0651	0.095*	
H13B	0.9070	0.3065	−0.0651	0.095*	
C14	0.8346 (3)	0.09313 (19)	−0.0469 (2)	0.0904 (9)	
H14A	0.8684	0.0352	−0.0678	0.108*	
H14B	0.7411	0.0974	−0.0681	0.108*	
C15	0.8511 (3)	0.09099 (15)	0.0481 (2)	0.0774 (8)	
H15A	0.8019	0.0385	0.0672	0.093*	
H15B	0.9439	0.0824	0.0695	0.093*	
C16	0.2317 (2)	0.19163 (14)	−0.02867 (12)	0.0491 (5)	
H16A	0.1688	0.2409	−0.0204	0.059*	
H16B	0.2117	0.1376	0.0045	0.059*	
C17	0.2202 (2)	0.16579 (16)	−0.12093 (13)	0.0588 (6)	
H17A	0.1306	0.1451	−0.1400	0.071*	
H17B	0.2379	0.2206	−0.1538	0.071*	
C18	0.4419 (2)	0.12244 (16)	−0.10682 (13)	0.0577 (6)	
H18A	0.4637	0.1760	−0.1401	0.069*	
H18B	0.5028	0.0721	−0.1157	0.069*	
C19	0.4594 (2)	0.14893 (13)	−0.01383 (13)	0.0511 (6)	
H19A	0.4426	0.0949	0.0203	0.061*	
H19B	0.5495	0.1699	0.0033	0.061*	
C20	0.2886 (2)	0.38543 (13)	0.16874 (12)	0.0491 (6)	
H20A	0.3701	0.4197	0.1852	0.059*	
H20B	0.2805	0.3373	0.2109	0.059*	
C21	0.1725 (2)	0.45096 (14)	0.16374 (15)	0.0614 (6)	
H21A	0.0914	0.4155	0.1494	0.074*	
H21B	0.1695	0.4795	0.2188	0.074*	
C22	0.1873 (3)	0.48121 (16)	0.02166 (15)	0.0686 (7)	
H22A	0.1942	0.5305	−0.0196	0.082*	
H22B	0.1067	0.4463	0.0040	0.082*	
C23	0.3039 (2)	0.41675 (13)	0.02300 (13)	0.0537 (6)	
H23A	0.3060	0.3894	−0.0327	0.064*	
H23B	0.3855	0.4514	0.0383	0.064*	

### Atomic displacement parameters (Å^2^)

**Table:**

	*U*^11^	*U*^22^	*U*^33^	*U*^12^	*U*^13^	*U*^23^
N1	0.0419 (10)	0.0561 (11)	0.0486 (11)	0.0033 (9)	−0.0009 (9)	0.0031 (9)
N2	0.0502 (12)	0.0428 (10)	0.0761 (14)	0.0041 (9)	0.0118 (10)	0.0083 (9)
N3	0.0421 (10)	0.0395 (9)	0.0387 (10)	0.0038 (8)	0.0082 (8)	−0.0013 (7)
N4	0.0493 (11)	0.0361 (9)	0.0369 (9)	0.0081 (8)	0.0094 (8)	0.0025 (7)
O1	0.0517 (10)	0.0513 (9)	0.0712 (11)	−0.0052 (7)	0.0115 (8)	0.0084 (8)
O2	0.0714 (13)	0.0905 (13)	0.1004 (15)	0.0057 (11)	0.0289 (11)	−0.0122 (11)
O3	0.0659 (11)	0.0584 (9)	0.0510 (9)	−0.0035 (8)	0.0136 (8)	−0.0120 (7)
O4	0.0982 (14)	0.0441 (9)	0.0708 (11)	0.0230 (9)	0.0155 (10)	0.0018 (8)
C1	0.0480 (13)	0.0383 (11)	0.0353 (11)	0.0058 (9)	0.0071 (10)	0.0006 (9)
C2	0.0505 (14)	0.0431 (12)	0.0454 (13)	0.0036 (10)	0.0093 (10)	0.0023 (10)
C3	0.0735 (17)	0.0400 (12)	0.0493 (13)	0.0079 (11)	0.0189 (13)	0.0099 (10)
C4	0.0773 (19)	0.0588 (14)	0.0523 (15)	0.0212 (14)	0.0189 (14)	0.0196 (12)
C5	0.0644 (17)	0.0677 (15)	0.0492 (14)	0.0214 (13)	0.0028 (12)	0.0088 (12)
C6	0.0518 (14)	0.0449 (12)	0.0413 (12)	0.0088 (10)	0.0061 (11)	0.0027 (10)
C7	0.092 (2)	0.0689 (16)	0.0790 (18)	−0.0034 (15)	0.0303 (16)	0.0272 (14)
C8	0.0450 (13)	0.0410 (12)	0.0468 (13)	0.0047 (10)	0.0085 (11)	−0.0034 (10)
C9	0.0424 (12)	0.0387 (11)	0.0357 (11)	0.0042 (9)	0.0054 (9)	0.0025 (9)
C10	0.0501 (15)	0.0861 (17)	0.0581 (15)	0.0003 (13)	−0.0069 (12)	−0.0005 (13)
C11	0.0475 (15)	0.0740 (16)	0.087 (2)	0.0135 (13)	0.0007 (14)	0.0196 (14)
C12	0.0577 (16)	0.0464 (13)	0.089 (2)	0.0013 (11)	0.0130 (14)	0.0057 (12)
C13	0.080 (2)	0.0721 (18)	0.089 (2)	0.0104 (15)	0.0226 (17)	0.0107 (15)
C14	0.081 (2)	0.073 (2)	0.121 (3)	0.0015 (16)	0.029 (2)	−0.0298 (18)
C15	0.0638 (17)	0.0423 (14)	0.130 (3)	0.0052 (12)	0.0270 (17)	0.0000 (15)
C16	0.0449 (13)	0.0583 (13)	0.0444 (13)	−0.0028 (11)	0.0062 (10)	−0.0068 (10)
C17	0.0561 (15)	0.0720 (15)	0.0475 (14)	0.0010 (13)	0.0037 (12)	−0.0057 (11)
C18	0.0589 (16)	0.0608 (14)	0.0560 (15)	0.0041 (12)	0.0174 (12)	−0.0102 (11)
C19	0.0514 (14)	0.0501 (13)	0.0522 (14)	0.0074 (11)	0.0076 (11)	−0.0048 (10)
C20	0.0627 (15)	0.0422 (11)	0.0428 (12)	0.0040 (11)	0.0088 (11)	−0.0023 (9)
C21	0.0774 (18)	0.0490 (13)	0.0602 (15)	0.0120 (12)	0.0177 (13)	−0.0042 (11)
C22	0.089 (2)	0.0560 (14)	0.0606 (16)	0.0260 (13)	0.0103 (14)	0.0119 (12)
C23	0.0662 (16)	0.0461 (12)	0.0502 (13)	0.0121 (11)	0.0127 (12)	0.0117 (10)

### Geometric parameters (Å, º)

**Table:**

N1—C8	1.367 (2)	C10—H10A	0.9700
N1—C6	1.407 (2)	C10—H10B	0.9700
N1—C10	1.454 (3)	C11—H11A	0.9700
N2—C11	1.446 (3)	C11—H11B	0.9700
N2—C15	1.453 (3)	C12—C13	1.496 (3)
N2—C12	1.454 (2)	C12—H12A	0.9700
N3—C19	1.460 (2)	C12—H12B	0.9700
N3—C16	1.462 (2)	C13—H13A	0.9700
N3—C9	1.471 (2)	C13—H13B	0.9700
N4—C20	1.460 (2)	C14—C15	1.498 (4)
N4—C9	1.463 (2)	C14—H14A	0.9700
N4—C23	1.471 (2)	C14—H14B	0.9700
O1—C8	1.218 (2)	C15—H15A	0.9700
O2—C14	1.411 (3)	C15—H15B	0.9700
O2—C13	1.415 (3)	C16—C17	1.502 (3)
O3—C18	1.415 (2)	C16—H16A	0.9700
O3—C17	1.423 (2)	C16—H16B	0.9700
O4—C21	1.416 (2)	C17—H17A	0.9700
O4—C22	1.425 (3)	C17—H17B	0.9700
C1—C2	1.377 (3)	C18—C19	1.514 (3)
C1—C6	1.387 (3)	C18—H18A	0.9700
C1—C9	1.522 (2)	C18—H18B	0.9700
C2—C3	1.400 (3)	C19—H19A	0.9700
C2—H2A	0.9300	C19—H19B	0.9700
C3—C4	1.379 (3)	C20—C21	1.500 (3)
C3—C7	1.510 (3)	C20—H20A	0.9700
C4—C5	1.389 (3)	C20—H20B	0.9700
C4—H4A	0.9300	C21—H21A	0.9700
C5—C6	1.380 (3)	C21—H21B	0.9700
C5—H5A	0.9300	C22—C23	1.499 (3)
C7—H7A	0.9600	C22—H22A	0.9700
C7—H7B	0.9600	C22—H22B	0.9700
C7—H7C	0.9600	C23—H23A	0.9700
C8—C9	1.561 (3)	C23—H23B	0.9700
C10—C11	1.518 (3)		
			
C8—N1—C6	111.35 (17)	O2—C13—C12	112.0 (2)
C8—N1—C10	122.82 (18)	O2—C13—H13A	109.2
C6—N1—C10	125.73 (18)	C12—C13—H13A	109.2
C11—N2—C15	112.30 (19)	O2—C13—H13B	109.2
C11—N2—C12	113.09 (19)	C12—C13—H13B	109.2
C15—N2—C12	108.31 (18)	H13A—C13—H13B	107.9
C19—N3—C16	109.01 (15)	O2—C14—C15	111.7 (2)
C19—N3—C9	114.25 (15)	O2—C14—H14A	109.3
C16—N3—C9	113.90 (14)	C15—C14—H14A	109.3
C20—N4—C9	114.50 (15)	O2—C14—H14B	109.3
C20—N4—C23	108.83 (14)	C15—C14—H14B	109.3
C9—N4—C23	115.55 (14)	H14A—C14—H14B	107.9
C14—O2—C13	110.01 (18)	N2—C15—C14	110.5 (2)
C18—O3—C17	109.76 (15)	N2—C15—H15A	109.6
C21—O4—C22	109.72 (15)	C14—C15—H15A	109.6
C2—C1—C6	119.68 (18)	N2—C15—H15B	109.6
C2—C1—C9	131.34 (19)	C14—C15—H15B	109.6
C6—C1—C9	108.97 (17)	H15A—C15—H15B	108.1
C1—C2—C3	120.5 (2)	N3—C16—C17	108.96 (16)
C1—C2—H2A	119.8	N3—C16—H16A	109.9
C3—C2—H2A	119.8	C17—C16—H16A	109.9
C4—C3—C2	118.1 (2)	N3—C16—H16B	109.9
C4—C3—C7	121.5 (2)	C17—C16—H16B	109.9
C2—C3—C7	120.4 (2)	H16A—C16—H16B	108.3
C3—C4—C5	122.7 (2)	O3—C17—C16	111.33 (18)
C3—C4—H4A	118.6	O3—C17—H17A	109.4
C5—C4—H4A	118.6	C16—C17—H17A	109.4
C6—C5—C4	117.5 (2)	O3—C17—H17B	109.4
C6—C5—H5A	121.2	C16—C17—H17B	109.4
C4—C5—H5A	121.2	H17A—C17—H17B	108.0
C5—C6—C1	121.5 (2)	O3—C18—C19	111.97 (17)
C5—C6—N1	128.3 (2)	O3—C18—H18A	109.2
C1—C6—N1	110.19 (17)	C19—C18—H18A	109.2
C3—C7—H7A	109.5	O3—C18—H18B	109.2
C3—C7—H7B	109.5	C19—C18—H18B	109.2
H7A—C7—H7B	109.5	H18A—C18—H18B	107.9
C3—C7—H7C	109.5	N3—C19—C18	108.84 (17)
H7A—C7—H7C	109.5	N3—C19—H19A	109.9
H7B—C7—H7C	109.5	C18—C19—H19A	109.9
O1—C8—N1	125.00 (19)	N3—C19—H19B	109.9
O1—C8—C9	126.93 (18)	C18—C19—H19B	109.9
N1—C8—C9	108.04 (17)	H19A—C19—H19B	108.3
N4—C9—N3	107.45 (15)	N4—C20—C21	108.70 (17)
N4—C9—C1	112.29 (14)	N4—C20—H20A	109.9
N3—C9—C1	115.03 (15)	C21—C20—H20A	109.9
N4—C9—C8	112.91 (15)	N4—C20—H20B	109.9
N3—C9—C8	107.82 (14)	C21—C20—H20B	109.9
C1—C9—C8	101.32 (16)	H20A—C20—H20B	108.3
N1—C10—C11	112.09 (19)	O4—C21—C20	112.16 (18)
N1—C10—H10A	109.2	O4—C21—H21A	109.2
C11—C10—H10A	109.2	C20—C21—H21A	109.2
N1—C10—H10B	109.2	O4—C21—H21B	109.2
C11—C10—H10B	109.2	C20—C21—H21B	109.2
H10A—C10—H10B	107.9	H21A—C21—H21B	107.9
N2—C11—C10	113.24 (18)	O4—C22—C23	112.10 (19)
N2—C11—H11A	108.9	O4—C22—H22A	109.2
C10—C11—H11A	108.9	C23—C22—H22A	109.2
N2—C11—H11B	108.9	O4—C22—H22B	109.2
C10—C11—H11B	108.9	C23—C22—H22B	109.2
H11A—C11—H11B	107.7	H22A—C22—H22B	107.9
N2—C12—C13	109.5 (2)	N4—C23—C22	108.35 (17)
N2—C12—H12A	109.8	N4—C23—H23A	110.0
C13—C12—H12A	109.8	C22—C23—H23A	110.0
N2—C12—H12B	109.8	N4—C23—H23B	110.0
C13—C12—H12B	109.8	C22—C23—H23B	110.0
H12A—C12—H12B	108.2	H23A—C23—H23B	108.4
			
C6—C1—C2—C3	−1.5 (3)	C2—C1—C9—C8	175.79 (18)
C9—C1—C2—C3	179.17 (18)	C6—C1—C9—C8	−3.60 (19)
C1—C2—C3—C4	0.3 (3)	O1—C8—C9—N4	−55.5 (2)
C1—C2—C3—C7	178.40 (18)	N1—C8—C9—N4	122.73 (16)
C2—C3—C4—C5	0.4 (3)	O1—C8—C9—N3	63.0 (2)
C7—C3—C4—C5	−177.7 (2)	N1—C8—C9—N3	−118.73 (16)
C3—C4—C5—C6	0.1 (3)	O1—C8—C9—C1	−175.82 (18)
C4—C5—C6—C1	−1.4 (3)	N1—C8—C9—C1	2.43 (18)
C4—C5—C6—N1	176.15 (19)	C8—N1—C10—C11	−95.5 (2)
C2—C1—C6—C5	2.1 (3)	C6—N1—C10—C11	80.6 (2)
C9—C1—C6—C5	−178.48 (18)	C15—N2—C11—C10	−164.15 (18)
C2—C1—C6—N1	−175.87 (16)	C12—N2—C11—C10	72.9 (2)
C9—C1—C6—N1	3.6 (2)	N1—C10—C11—N2	59.7 (3)
C8—N1—C6—C5	−179.7 (2)	C11—N2—C12—C13	−176.73 (18)
C10—N1—C6—C5	3.8 (3)	C15—N2—C12—C13	58.1 (2)
C8—N1—C6—C1	−2.0 (2)	C14—O2—C13—C12	57.4 (3)
C10—N1—C6—C1	−178.44 (18)	N2—C12—C13—O2	−59.1 (3)
C6—N1—C8—O1	177.82 (17)	C13—O2—C14—C15	−56.3 (3)
C10—N1—C8—O1	−5.6 (3)	C11—N2—C15—C14	176.5 (2)
C6—N1—C8—C9	−0.5 (2)	C12—N2—C15—C14	−57.9 (3)
C10—N1—C8—C9	176.11 (17)	O2—C14—C15—N2	57.9 (3)
C20—N4—C9—N3	174.99 (14)	C19—N3—C16—C17	−59.6 (2)
C23—N4—C9—N3	−57.4 (2)	C9—N3—C16—C17	171.50 (16)
C20—N4—C9—C1	47.5 (2)	C18—O3—C17—C16	−58.4 (2)
C23—N4—C9—C1	175.20 (17)	N3—C16—C17—O3	59.8 (2)
C20—N4—C9—C8	−66.3 (2)	C17—O3—C18—C19	57.7 (2)
C23—N4—C9—C8	61.4 (2)	C16—N3—C19—C18	58.5 (2)
C19—N3—C9—N4	175.91 (14)	C9—N3—C19—C18	−172.81 (15)
C16—N3—C9—N4	−57.90 (19)	O3—C18—C19—N3	−58.3 (2)
C19—N3—C9—C1	−58.3 (2)	C9—N4—C20—C21	−169.33 (16)
C16—N3—C9—C1	67.9 (2)	C23—N4—C20—C21	59.7 (2)
C19—N3—C9—C8	53.93 (19)	C22—O4—C21—C20	57.3 (3)
C16—N3—C9—C8	−179.89 (15)	N4—C20—C21—O4	−59.2 (2)
C2—C1—C9—N4	55.1 (3)	C21—O4—C22—C23	−57.4 (3)
C6—C1—C9—N4	−124.33 (17)	C20—N4—C23—C22	−59.6 (2)
C2—C1—C9—N3	−68.2 (2)	C9—N4—C23—C22	169.92 (18)
C6—C1—C9—N3	112.37 (18)	O4—C22—C23—N4	59.0 (2)

### Hydrogen-bond geometry (Å, º)

**Table:**

*D*—H···*A*	*D*—H	H···*A*	*D*···*A*	*D*—H···*A*
C20—H20*B*···O3^i^	0.97	2.62	3.109 (3)	112
C15—H15*A*···O3^ii^	0.97	2.52	3.480 (2)	173
C17—H17*A*···O2^iii^	0.97	2.66	3.395 (3)	132
C13—H13*B*···O4^iv^	0.97	2.64	3.318 (3)	128

Symmetry codes: (i) *x*, −*y*+1/2, *z*+1/2; (ii) −*x*+1, −*y*, −*z*; (iii) *x*−1, *y*, *z*; (iv) −*x*+1, −*y*+1, −*z*.
